# Selection and Validation of Reference Genes for Reverse-Transcription Quantitative PCR Analysis in *Sclerotium rolfsii*

**DOI:** 10.3390/ijms242015198

**Published:** 2023-10-15

**Authors:** Chaofan Jiang, Lin Zhou, Qingchen Zhao, Mengke Wang, Sirui Shen, Te Zhao, Kaidi Cui, Leiming He

**Affiliations:** 1 College of Plant Protection, Henan Agricultural University, Zhengzhou 450046, China; 2 Henan Key Laboratory of Creation and Application of New Pesticide, Henan Agricultural University, No. 218, Ping’an Avenue, Zhengzhou 450046, China; 3 Henan Research Center of Green Pesticide Engineering and Technology, Henan Agricultural University, Zhengzhou 450046, China

**Keywords:** *Sclerotium rolfsii*, reference gene, RT-qPCR, expression stability

## Abstract

Reference genes are important for the accuracy of gene expression profiles using reverse-transcription quantitative PCR (RT-qPCR). However, there are no available reference genes reported for *Sclerotium rolfsii*; it actually has a pretty diverse and wide host range. In this study, seven candidate reference genes (*UBC*, *β-TUB*, *28S*, *18S*, *PGK*, *EF1α* and *GAPDH*) were validated for their expression stability in *S. rolfsii* under conditions of different developmental stages, populations, fungicide treatments, photoperiods and pHs. Four algorithm programs (geNorm, Normfinder, Bestkeeper and ΔCt) were used to evaluate the gene expression stability, and RefFinder was used to integrate the ranking results of four programs. Two reference genes were recommended by RefFinder for RT-qPCR normalization in *S. rolfsii*. The suitable reference genes were *GAPDH* and *UBC* across developmental stages, *PGK* and *UBC* across populations, *GAPDH* and *PGK* across fungicide treatments, *EF1α* and *PGK* across photoperiods, *β-TUB* and *EF1α* across pHs and *PGK* and *GAPDH* across all samples. Four target genes (*atrB*, *PacC*, *WC1* and *CAT*) were selected for the validation of the suitability of selected reference genes. However, using one or two reference genes in combination to normalize the expression of target genes showed no significant difference in *S. rolfsii*. In short, this study provided reliable reference genes for studying the expression and function of genes in *S. rolfsii*.

## 1. Introduction

Reverse-transcription quantitative PCR (RT-qPCR) is a popular and reliable tool for quantification of gene expression profiles due to its rapidity, wide dynamic range, sensitivity and specificity [1,2]. However, the accuracy of RT-qPCR relies on several factors, including RNA quality, reverse transcription efficiency, PCR reaction and data analysis [3]. To overcome the unavoidable experimental bias, normalization with reference genes that are stably expressed under different environmental conditions is a necessary and efficient method to increase the accuracy of RT-qPCR analysis.

Frequently used reference genes in RT-qPCR assays include glyceraldehyde-3-phosphate dehydrogenase (*GAPDH*), 18S ribosomal RNA, actin (*ACT*) and tubulin (*TUB*), as they are stably expressed in some tissues/organisms under certain treatment conditions [2,4]. However, the expression of widely used reference genes are not consistently stable among different stress conditions, developmental stages and multiple species [5]. The arbitrary use of reference genes can hide the true differences in gene expression between samples, resulting in inaccurate RT-qPCR results [6]. It has been reported that selecting different reference genes can produce a 100-fold variation in the expression of the same target gene [7]. Therefore, it is critical to select and validate suitable reference genes in each species for different experimental conditions.

To date, there are no reported appropriate and reliable reference genes identified in *Sclerotium rolfsii* (Teleomorph: *Aathelia rolfsii*), the causal pathogen of plant stem rot, which can cause diseases in more than 500 plants and leads to huge economic losses for the world [8]. *Sclerotium rolfsii* initially produces white silky mycelia on the plant stems, and then forms a dark brown sclerotia that spreads with agricultural operations or remains on plant debris and in soil for several years [9]. *Sclerotium rolfsii* can be divided into different mycelial compatibility groups (MCGs) based on mycelial interactions [10]. Generally, the application of fungicides is the common control method for the management of this disease [9,11]. The development, survival and genetic variability of *S. rolfsii*, as well as its response to fungicides, are inseparable from the regulation of genes. The validation of suitable reference genes is of great importance to analyze gene functions and verify the gene expression patterns in *S. rolfsii* under different abiotic and biotic environmental conditions.

In this study, to identify suitable reference genes for normalization of gene expression in *S. rolfsii*, seven candidate reference genes, including ubiquitin-conjugating enzyme (*UBC*), β-tubulin (*β-TUB*), eukaryotic 28S rRNA (*28S*), eukaryotic 18S rRNA (*18S*), 3-phosphoglycerate kinase (*PGK*), translation elongation factor 1-α (*EF1α*) and *GAPDH*, were selected according to the *S. rolfsii* RNA-seq data and examined for their expression stability across differences in developmental stage, population, fungicide treatment, photoperiod and pH. The expression stability of candidate reference genes was evaluated and ranked by four algorithms geNorm (v 3.5) [12], NormFinder (v 0.953) [13], BestKeeper (v 1) [14] and ΔCt [15] and RefFinder software package (http://blooge.cn/RefFinder/, accessed on 1 May 2023). In addition, four stress-induced target genes, including ATP-binding cassette transporter atrB (*atrB*), pH-response transcription factor (*PacC*), white collar-1 (*WC1*) and catalase (*CAT*), were used to validate the of suitability of the candidate reference genes. This study could provide reliable reference genes in *S. rolfsii* to facilitate future gene expression studies in this species.

## 2. Results

### 2.1. Primer Specificity and Amplification Efficiency

The 2% agarose gel electrophoresis analysis of PCR products clearly showed single bands of predicted sizes, while primer dimers and non-specific amplification were not detected in the experiment (Figure S2A). In addition, single peaks were obtained in the melting curve analysis (Figure S2B), indicating the high amplification specificity of the designed primers. The amplification efficiency of all primers ranged from 98.26% to 109.20%, and the correlation coefficients (*R*^2^) of all linear regressions were higher than 0.9901 (Table 1).

### 2.2. Expression Levels of the Candidate Reference Genes

**Figure 1:**
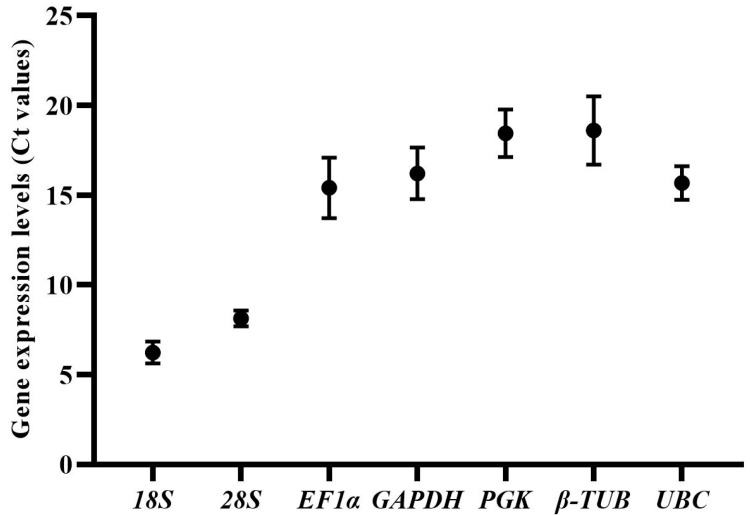
Expression levels (Ct values) of the seven candidate reference genes in *S. rolfsii* under all experimental conditions. The bars represent the standard deviation.

The median expression levels (Ct values) of seven candidate reference genes in all samples were below 20 (Table S1, Figure 1). *18S* has the lowest Ct value and standard deviation (6.24 ± 0.61), showing that the expression of *18S* was high and most stable in all samples. The expression level order of genes was as follows: *18S* (with Ct value of 6.24 ± 0.61) > *28S* (8.13 ± 0.44) > *EF1*α (15.41 ± 1.69) > *UBC* (15.68 ± 0.93) > *GAPDH* (16.22 ± 1.44) > *PGK* (18.45 ± 1.33) > *β-TUB* (18.61 ± 1.90). Each reference gene had variations in the expression levels of samples. *18S* and *28S* had the lowest expression variations among all samples, while *β-TUB*, *EF1*α and *GAPDH* showed the highest expression variations. However, it should be noted that the expression of most candidate reference genes was stable in mycelia samples, the expression variations of genes (including *β-TUB*, *EF1*α, *GAPDH*, *PGK* and *UBC*) mainly existed in the samples of different developmental stages (mycelia and sclerotia).

### 2.3. Expression Stability of the Candidate Reference Genes

#### 2.3.1. Developmental Stages

**Figure 2:**
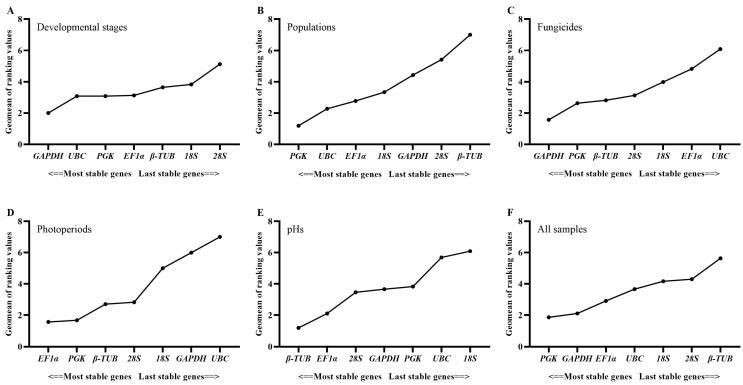
The expression stability order of seven candidate reference genes in *S. rolfsii* according to RefFinder across conditions of (**A**) developmental stages (mycelia and sclerotia), (**B**) populations (three MCGs), (**C**) fungicides (tebuconazole, prothioconazole, thifluzamide, carboxin and azoxystrobin), (**D**) photoperiods (24 h of dark and light), (**E**) pHs (5, 7 and 9), and (**F**) all samples. For (**B**–**E**), mycelia were used.

**Figure 3:**
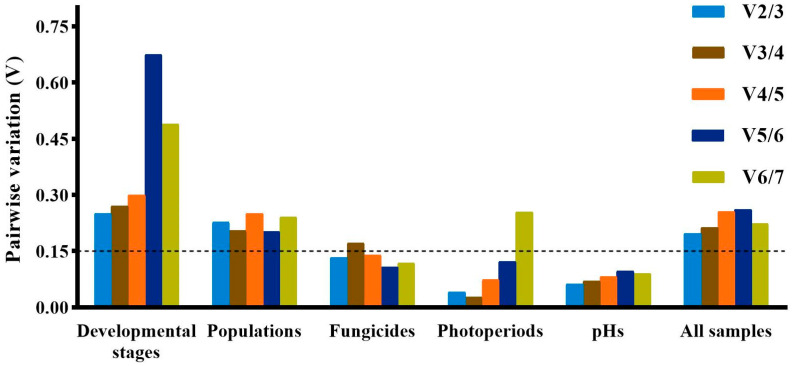
Pairwise variation (V) analysis of seven candidate reference genes calculated by geNorm under different experimental conditions. A cut-off value of 0.15 was used to determine the optimal number of reference genes.

The geNorm, NormFinder and ΔCt method identified *28S* and *18S* as the least stably expressed genes across developmental stages, while Bestkeeper identified *β-TUB* and *EF1α* as the least stably expressed genes. The top three stably expressed genes were *EF1α*, *β-TUB* and *PGK* according to geNorm; *GAPDH*, *UBC* and *PGK* according to Normfinder; *18S*, *28S* and *UBC* according to Bestkeeper; and *GAPDH*, *PGK* and *UBC* according to the ΔCt method (Table 2). RefFinder ranked the stability of genes in the following order: *GADPH* > *UBC* > *PGK* > *EF1α* > *β-TUB* > *18S* > *28S* (Figure 2). All the pairwise variation values in the geNorm analysis were above the cut-off of 0.15 (Figure 3). In this case, the top two or three stably expressed genes are usually selected according to the geNorm manual. Therefore, *GADPH* and *UBC* were recommended as reference genes across developmental stages (Table 3).

#### 2.3.2. Populations

*PGK*, *EF1α* and *UBC* were identified as the genes with the most stable expression by all the algorithms, except Bestkeeper, which identified *18S*, *PGK* and *UBC* as the three best stably expressed genes (Table 2). All four algorithms identified *β-TUB* as the least stably expressed gene. The stability order of genes ranked by RefFinder was *PGK* > *UBC* > *EF1α* > *18S* > *GADPH* > *28S* > *β-TUB* (Figure 2). All the pairwise variation values in the geNorm analysis were above the cut-off of 0.15 (Figure 3). *PGK* and *UBC* were recommended as the reference genes across populations (Table 3).

#### 2.3.3. Fungicides

According to Normfinder and the ΔCt method, the top three stably expressed genes were *GAPDH*, *PGK* and *β-TUB*, and the least stably expressed genes were *UBC* and *EF1α* (Table 2). Based on geNorm, *GAPDH*, *β-TUB* and *EF1α* were regarded as the most stably expressed genes, while *UBC* and *28S* were the least stably expressed genes. Bestkeeper identified *28S*, *18S* and *PGK* as the top three stably expressed genes, while *β-TUB* and *GAPDH* were the least stably expressed genes. The order from the most stable to least stable genes in the RefFinder analysis was *GAPDH* > *PGK* > *β-TUB* > *28S* > *18S* > *EF1α* > *UBC* (Figure 2). The geNorm analysis showed that pairwise variation values of V2/3, V4/5, V5/6 and V6/7 were less than 0.15 (Figure 3). Based on the convenience and cost, two reference genes (*GAPDH* and *PGK*) were recommended as suitable reference genes across the tested fungicide treatments (Table 3).

#### 2.3.4. Photoperiods

Across photoperiod treatments, geNorm, Normfinder and the ΔCt method regarded *PGK*, *EF1α* and *β-TUB* as the most stably expressed genes, while Bestkeeper regarded *28S*, *β-TUB* and *EF1α* as the most stably expressed genes (Table 2). All four algorithms identified *UBC* and *GAPDH* as the least stably expressed genes. According to RefFinder, the stability order of gene expression was *EF1α* > *PGK* > *β-TUB* > *28S* > *18S* > *GAPDH* > *UBC* (Figure 2). The geNorm analysis showed that V2/3, V3/4, V4/5 and V5/6 values were less than 0.15 (Figure 3). Therefore, *EF1α* and *PGK* were considered as the most suitable reference genes to normalize RT-qPCR data from different photoperiod samples (Table 3).

#### 2.3.5. pHs

Both geNorm and the ΔCt method identified same stably expressed genes (*β-TUB*, *EF1α* and *PGK)* and least stably expressed genes (*18S* and *GAPDH*) (Table 2). However, the top three stably expressed genes identified by Normfinder were *β-TUB*, *EF1α* and *28S*, and the two least stably expressed genes were *18S* and *UBC*. According to Bestkeeper, the top three stably expressed genes were *GAPDH*, *β-TUB* and *28S*, and the two least stably expressed genes were *UBC* and *PGK*. Based on RefFinder, the stability order of gene expression was *β-TUB* > *EF1α* > *28S* > *GAPDH* > *PGK* > *UBC* > *18S* (Figure 2). All pairwise variation values in geNorm analysis were less than 0.15 (Figure 3). Therefore, *β-TUB* and *EF1α* were recommended as reference genes to normalize gene expression under different pH conditions (Table 3).

#### 2.3.6. Ranking of Reference Genes for All Samples

Across all samples, the most stably expressed genes were *PGK, GAPDH* and *EF1α* according to geNorm and the ΔCt method; *PGK*, *GAPDH* and *UBC* according to Normfinder; and *28S*, *18S* and *UBC* according to Bestkeeper (Table 2). The two most unstably expressed genes were *28S* and *β-TUB* according to Normfinder and the ΔCt method; *28S* and *18S* according to geNorm; and *β-TUB* and *EF1α* according to Bestkeeper (Table 2). Based on RefFinder, the stability order of gene expression across all samples was *PGK* > *GAPDH* > *EF1α* > *UBC* > *18S* > *28S* > *β-TUB* (Figure 2). The geNorm analysis showed that all pairwise variation values were greater than 0.15 (Figure 3). Therefore, *PGK* and *GAPDH* were considered as the most suitable reference genes for RT-qPCR (Table 3).

#### 2.3.7. Reference Gene Validation

**Figure 4:**
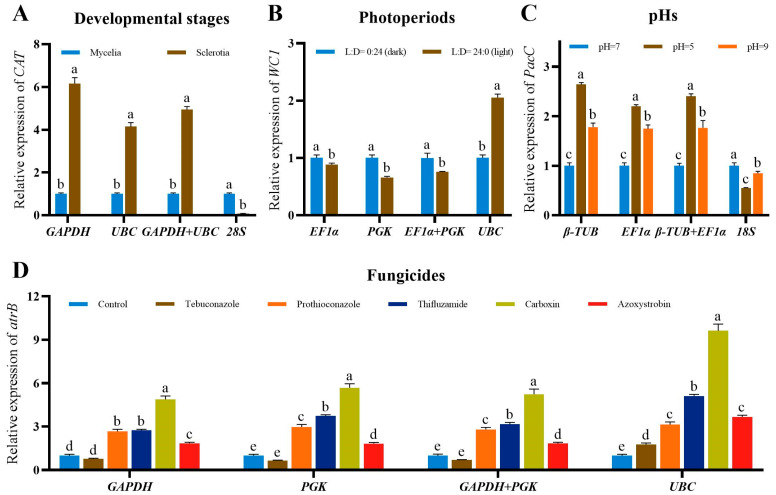
Relative expression of target genes in *S. rolfsii* at different experimental conditions. (**A**) Expression profiles of *CAT* at different developmental stages. (**B**) Expression profiles of *WC1* under different photoperiods. (**C**) Expression profiles of *PacC* under different pHs. (**D**) Expression profiles of *atrB* under different fungicide treatments. The expression profiles of target genes were normalized by the two most stably expressed (single and in combination) and the least stably expressed reference genes recommend by RefFinder. Values are means ± SD (*n* = 3). Different letters on the bars indicate significant differences between different treatments (*p* < 0.05, Tukey’s HSD test).

When the most two stably expressed reference genes were used alone or in combination to normalize the expression of target genes (*CAT*, *WC1*, *PacC* and *atrB)* under different developmental stages, photoperiods, pHs and fungicide treatments, the expression of target genes showed similar trends with minor differences (Figure 4). In comparison, the expressions of target genes were obviously different when the unstably expressed gene was used as the reference gene to normalize RT-qPCR data. For example, the expressions of *CAT* using *GAPDH*, *UBC* or *GAPDH*+*UBC* as normalizers were consistent and different to those using the unstably expressed gene *28S* as the normalizer (Figure 4A).

## 3. Discussion

RT-qPCR is a widely used technique to quantify the gene expression profiles in species [16]. At present, suitable reference genes have been screened and validated in many fungal species [17,18,19,20]. However, there are no available reference genes reported for *S. rolfsii*. This will inevitably hinder the study of gene functions and transcriptome verification of *S. rolfsii*. It is well known that, except for the selection of reference genes, the accuracy of RT-qPCR also depends on the RNA quality and amplification efficiency [3]. In this study, the A260/A280 ratios of all samples ranged from 1.8 to 2.0, and the amplification efficiency of all candidate genes were between 98.26% and 109.20%. Therefore, the RNA quality and amplification efficiency of samples were eligible for RT-qPCR.

In general, the expression of reference genes is not always stable under all stress conditions [6,21], which was consistent with our findings in *S. rolfsii*. It suggests that suitable reference genes should be chosen depending on the specific stress conditions and tissues or developmental stages. The suitability of reference genes needs to be validated with suitable target genes under specific stress conditions. It is known that sclerotia formation is triggered by oxidative stress, and reactive oxygen species (ROS) indicators such as CAT activity was upregulated in *S. rolfsii* and *S. sclerotiorum* [22]; WC proteins (WC1 and WC2) are important photoreceptor proteins in the light signal transduction in fungi [23,24]; the transcription factor PacC is a pH regulator and affects the virulence of fungi [25,26]; and gene *atrB* is usually overexpressed under fungicide treatments [27]. Therefore, genes *CAT*, *WC1*, *PacC* and *atrB* are chosen as the target genes in *S. rolfsii* in conditions of different developmental stages, photoperiods, pHs and fungicides.

Our results indicated that *GAPDH* was the most stably expressed internal control gene in *S. rolfsii* for RT-qPCR normalization under different developmental stages, fungicide treatments and all tested conditions. Previous studies also identified *GAPDH* as the recommended reference gene in different developmental stages of fungi *Sparassis latifolia* [28] and amphibian *Andrias davidianus* [29]. Additionally, *GAPDH* showed the best stability in *Pythium porphyrae* under conditions of salinity, pH and infection stages [30]. As far as we know, there are only a few reports selecting and validating the suitable reference genes to compare the gene expression between the mycelia and sclerotia of pathogens, such as *S. sclerotiorum* and *Rhizoctonia solani*. Whether *GAPDH* could be used as normalizers in other pathogens that produce sclerotia should be further validated. Figure 4 suggested that randomly using an unstably expressed reference gene to normalize RT-qPCR data could generate inaccurate interpretation of gene expression differences. In this study, two reference genes were recommended by RefFinder for RT-qPCR normalization in *S. rolfsii*. *UBC* and *PGK* was also recommended as a reference gene in *S. rolfsii* for RT-qPCR analysis respect to different developmental stages and fungicide treatments, respectively. However, using one or two reference genes in combination to normalize the expression of target genes showed no significant difference in *S. rolfsii*. Therefore, based on the convenience and cost, one reference gene was enough for RT-qPCR normalization in *S. rolfsii*.

*EF1*α is a GTP-binding protein to catalyze the combining of aminoacyl-transfer RNAs to the ribosome [31]. It is highly conserved among species and ranked first among internal control genes in the ICG website [32]. In current study, *EF1*α was an ideal reference gene in *S. rolfsii* subjected to photoperiod and pH treatments. *EF1*α is also commonly used as a stably expressed reference gene in many species under various conditions, such as *Mythimna separata* under different photoperiod treatments [33], *Pythium porphyrae* under different pH treatments [30], *Athetis dissimilis* under different insecticide treatments [3] and *Glycine max* under different developmental stages and stress conditions [34]. Other than *EF1*α, *PGK* and *β-TUB* were also suitable reference genes in *S. rolfsii* across photoperiod and pH treatments, respectively. But, as mentioned earlier, using either reference gene was sufficient to ensure the accuracy of RT-qPCR results.

In addition to species and exposure conditions, the algorithms used also affect the stability ranking of reference genes. For example, in this study with *S. rolfsii*, the most stably expressed genes across fungicide treatments were *GAPDH*, *PGK* and *β-TUB* identified by Normfinder and the ΔCt method, *GAPDH*, *β-TUB* and *EF1α* by geNorm, and *28S*, *18S* and *PGK* by Bestkeeper. The inconsistencey of ranking between the four programs resulted from the differences in their statistical analyses. Normfinder and geNorm rank the stability of genes using the expression stability value (M-value) and stability value (SV), respectively; Bestkeeper compares the stability of genes using the coefficient of variation (CV) and standard deviation (SD); and the ΔCt method analyze the stability of genes using the average standard deviation (Avg. SD) [21]. Although using a multi-algorithm analysis could increase the accuracy of validation of reference genes, to avoid the experimental bias of these algorithms, the fourth program (RefFinder) was used to integrate the results of four algorithms and comprehensively evaluate the stability of seven candidate reference genes. According to RefFinder, the stability ranking of reference genes in *S. rolfsii* under all experimental conditions was *PGK* > *GAPDH* > *EF1α* > *UBC* > *18S* > *28S* > *β-TUB*.

In conclusion, the most stably expressed reference genes identified by RefFinder were *GAPDH* and *UBC* across developmental stages, *PGK* and *UBC* across populations, *GAPDH* and *PGK* under different fungicide treatments, *EF1α* and *PGK* under different photoperiods, *β-TUB* and *EF1α* under different pHs and *PGK* and *GAPDH* under all conditions. Using an unstably expressed reference gene to normalize RT-qPCR data could generate inaccurate interpretation of gene expression differences. However, using one or two reference genes in combination to normalize the expression of target genes showed no significant difference in *S. rolfsii*. Our results highlighted the importance of selecting suitable reference genes for RT-qPCR analysis and provided useful information for the gene expression studies of *S. rolfsii* in future.

## 4. Materials and Methods

### 4.1. Strains

*S. rolfsii* strains ZZ-3, SR-7 and HX-5 were collected from different peanut planting regions in China (Xinxiang, Henan; Rizhao, Shandong; and Xingtai, Hebei). Three strains were isolated and identified by morphological and molecular biological analysis according to the method described by Han et al. [9]. *Sclerotium rolfsii* was cultured on potato dextrose agar (PDA) plates (90 mm) and incubated at 28 °C in the dark.

### 4.2. Experimental Conditions

Factors, including developmental stage, population, fungicide treatment, photoperiod and pH, would affect the expression of candidate reference genes. *Sclerotium rolfsii* samples after treatments with above factors were collected, frozen in liquid nitrogen and stored at −80 °C for further study. Each treatment contained three independent replicates, and each experiment was replicated three times.

#### 4.2.1. Developmental Stages

Fresh mycelial plugs (5 mm) of *S. rolfsii* (ZZ-3) were transferred on the center of cellophane-overlaid PDA plates and incubated at 28 °C in the dark. After incubation for 2 days, the mycelia were collected. At the same time, mycelial plugs of *S. rolfsii* were cultured on PDA plates for a month to produce sclerotia. Each developmental stage of *S. rolfsii* sample (0.1 g of mycelia and sclerotia) was placed in a 1.5 mL Eppendorf tube, frozen and stored.

#### 4.2.2. Populations

According to the previous classification of *S. rolfsii* populations, strains ZZ-3, SR-7 and HX-5 belonged to different MCGs based on mycelial interactions (Figure S1). Fresh mycelial plugs (5 mm) of each isolate were placed on cellophane-overlaid PDA plates and cultured at 28 °C for 2 days in the dark. Mycelia were harvested, frozen and stored.

#### 4.2.3. Fungicides

Fresh mycelial plugs (5 mm) of strain ZZ-3 were cultured on cellophane-overlaid PDA plates, which were amended with the EC_50_ value of tebuconazole (0.03 mg/L), prothioconazole (0.1 mg/L), thifluzamide (0.05 mg/L), carboxin (0.2 mg/L) and azoxystrobin (0.3 mg/L). PDA plates amended without fungicides were used as controls. After incubation at 28 °C for 2 days in the dark, mycelia were harvested, frozen and stored.

#### 4.2.4. Photoperiods

Fresh mycelial plugs (5 mm) of strain ZZ-3 were cultured on cellophane-overlaid PDA plates under photoperiod (L:D) of 0:24 (dark) and 24:0 (light). After incubation at 28 °C for 2 days, mycelia were harvested, frozen and stored.

#### 4.2.5. pHs

The pH of PDA medium was regulated with 1% HCl and 1% NaOH solution to 5, 7 and 9. Fresh mycelial plugs (5 mm) of strain ZZ-3 were cultured on cellophane-overlaid PDA plates with different pHs. After incubation at 28 °C for 2 days in the dark, mycelia were harvested, frozen and stored.

### 4.3. Candidate Reference Genes and Primer Design

Seven housekeeping genes commonly used in other fungi, including *UBC*, *β-TUB*, *28S*, *18S*, *PGK*, *EF1α* and *GAPDH*, were chosen as candidate references genes to be assessed in *S. rolfsii*. The sequences of these genes were obtained from our *S. rolfsii* transcriptome data. The primer pairs were designed by Beacon Designer 7.9 (PREMIER Biosoft, San Francisco, CA, USA) and synthesized by Sangon Biotech Co., Ltd. (Shanghai, China). The parameters of the primers were as follows: primer length of 18–25 bp, amplified product length of 80–180 bp, melting temperature of 60 ± 3 °C, CG content of 45–55%. The information of all primers is listed in Table 1.

### 4.4. RNA Extraction, cDNA Synthesis and RT-qPCR

Total RNA was extracted from frozen *S. rolfsii* samples using RNAiso Plus regent (TaKaRa, Kusatsu, Japan) and treated with DNase I. One microgram of RNA was used to synthesize first-strand cDNA using PrimeScript™ RT reagent Kit with gDNA Eraser (TaKaRa, Japan). The RT-qPCR reaction was carried out using PowerUp SYBR Green PCR Master Mix (Thermo Fisher Scientific, Waltham, MA, USA) on the Applied Biosystems QuantStudio 3 Real-Time PCR System (Thermo Fisher Scientific). Each 20 μL reaction system contained 10 μL of 2 × PowerUp SYBR Green Master Mix, 1 μL of each forward and reverse primer (10 μM), 1 μL of cDNA, and 7 μL of ddH_2_O. Cycling system was performed at 95 °C for 30 s, followed by 40 cycles of 95 °C for 10 s and 60 °C for 10 s. The specificity of RT-qPCR products was verified by a melting curve analysis from 65 °C to 95 °C. Three biological replicates with three technical replicates were set up for each treatment in RT-qPCR analysis. A series of diluted cDNAs (1×, 10×, 100×, 1000×, 10,000×) were used to construct a standard curve to calculate its correlation coefficient and slope value. The amplification efficiency was calculated with the equation [10^(1/−slope)^ − 1] × 100%.

### 4.5. Gene Expression Stability Analysis

Four algorithms (geNorm, NormFinder, BestKeeper and ΔCt) were used to evaluate the expression stability of seven candidate reference genes across all treatments. The detailed calculation method of parameter values in each algorithm was described previously [21]. The lower the value obtained by the algorithms, the more stable the gene expression. RefFinder package (http://blooge.cn/RefFinder/, accessed on 1 May 2023) was used to combine the results of four algorithms and give a comprehensive stability ranking of candidate reference genes.

### 4.6. Suitability Validation

To validate the reliability of selected reference genes, four target genes, including white Collar-1 (*WC1*) for photoperiod treatment, pH-response transcription factor (*PacC*) for pH treatment, catalase (*CAT*) for different developmental stages and ATP-binding cassette transporter atrB (*atrB*) for fungicide treatments, were evaluated using two most stably expressed and the least stably expressed reference genes recommend by RefFinder. Since there are no studies reporting the differentially expressed genes between different MCGs of *S. rolfsii*, no suitable target genes were available for verifying the expression stability of reference genes among different populations. Therefore, the expression of target genes across populations was not evaluated. The relative gene expression levels of target genes were calculated using the 2^−ΔΔCt^ method [35]. Three biological replicates with three technical replicates were conducted for each treatment. The statistical analysis of the gene expression levels was performed using one-way ANOVA followed by Tukey’s HSD test (*p* < 0.05) through SPSS 18.0 software (SPSS Inc., Chicago, IL, USA).

## Figures and Tables

**Table 1:** Primer sequences and amplification characteristics of candidate genes used for RT-qPCR.

Symbol	Gene Name	Accession Number	Primer Sequence (5′-3′)	Amplification Efficiency (%)	Product Length (bp)	Tm	Correlation Coefficient (*R*^2^)
*UBC*	Ubiquitin-conjugating enzyme	OQ944110	F:AGGGTATTCCTCCCGACCAGCA R:CACGAAGACGGAGAACCAGATGAAG	105.42	123	63.1	0.9975
						59.7	
*β-TUB*	β-tubulin	OQ944109	F:GCTCAGCACGCCTACATACGG R:AGACGAGGGAAGGGCACCAT	109.20	141	61.4	0.9901
						61.7	
*28S*	28S ribosome	OQ944112	F:TCTACTTGTGCGCTATCGGTCTCT R:ACGAGTCGAGTTGTTTGGGAATGC	103.23	93	59.6	0.9987
						60.0	
*GAPDH*	Glyceraldehyde-3-phosphate dehydrogenase	OQ944106	F:ACCAAGTCATCTCCAACGCTTCCT R:ACCGCCACGCCAGTCCTT	102.82	167	60.6	0.9937
						63.7	
*18S*	18S ribosome	OQ944113	F:AGTTGGTGGAGTGATTTGTCTGGT R:CAGTCCCTCTAAGAAGCCAGCAATC	99.76	105	58.3	0.9952
						59.7	
*PGK*	3-Phosphoglycerate kinase	OQ944108	F:CGACAAGGACGCCACAACTG R:AAGCACGGTCTCACGGAACA	107.82	112	59.7	0.9996
						59.5	
*EF1α*	Elongation factor-1α	OQ944105	F:GCTTCCTTCAACGCTCAGGTCATC R:AATGTGGGCAGTGTGGCAATCAA	98.26	93	60.7	0.9969
						60.0	
*WC1*	White Collar-1	OQ944111	F:TATTGCCATACAGCGGATGTCGTG R:CGTGGGGGTGATGCGT	101.65	143	59.3	0.9988
						58.4	
*PacC*	pH-response transcription factor	OQ944107	F:CACATACAGCACAAGCACTCCAAGG R:AGCAGCAATAGCGGCGTTCT	106.46	99	60.6	0.9972
						60.2	
*CAT*	Catalase	OQ944104	F:CCGCAGAGACACACCAAGGATG R:GTGCCACGGTCACTGAAGAGAA	108.29	101	59.5	0.9994
						59.3	
*atrB*	ATP-binding cassette transporter atrB	OQ953998	F:GACACGCTCGTCAACGGAAG R:GCGTAGATGCTAATGGAGATGGACT	100.51	179	61.1	0.9978
						59.9	

**Table 2:** Expression stability of seven candidate reference genes in *S. rolfsii* under different experimental conditions.

Conditions	Reference Gene	geNorm		Normfinder		Bestkeeper		ΔCt	
		M	Rank	SV	Rank	CV + SD	Rank	Avg. SD	Rank
Developmental stages	*18S*	2.07	5	3.28	6	0.35	1	3.38	6
	*28S*	2.45	6	3.34	7	0.39	2	3.42	7
	*EF1α*	0.04	1	2.12	4	3.08	6	2.30	4
	*GAPDH*	0.80	3	0.29	1	2.15	4	1.84	1
	*PGK*	0.51	2	1.11	3	2.56	5	1.94	2
	*β-TUB*	0.04	1	2.17	5	3.11	7	2.34	5
	*UBC*	1.07	4	0.34	2	1.67	3	1.95	3
Populations	*18S*	0.93	4	1.02	5	0.38	1	1.30	5
	*28S*	1.04	5	1.35	6	0.51	4	1.48	6
	*EF1α*	0.51	2	0.39	2	0.77	5	1.01	2
	*GAPDH*	0.67	3	0.72	4	0.90	6	1.14	4
	*PGK*	0.16	1	0.33	1	0.40	2	0.97	1
	*β-TUB*	1.25	6	1.65	7	1.44	7	1.76	7
	*UBC*	0.16	1	0.58	3	0.44	3	1.05	3
Fungicides	*18S*	0.65	4	0.58	5	0.46	2	0.77	5
	*28S*	0.68	5	0.51	4	0.27	1	0.74	4
	*EF1α*	0.40	2	0.62	6	0.77	5	0.78	6
	*GAPDH*	0.33	1	0.34	1	0.78	6	0.64	1
	*PGK*	0.56	3	0.41	2	0.57	3	0.69	2
	*β-TUB*	0.33	1	0.49	3	0.78	7	0.69	3
	*UBC*	0.75	6	0.80	7	0.64	4	0.91	7
Photoperiods	*18S*	0.21	4	0.38	5	0.48	5	0.64	5
	*28S*	0.10	3	0.02	4	0.16	1	0.53	4
	*EF1α*	0.03	1	0.01	2	0.26	3	0.50	1
	*GAPDH*	0.39	5	0.97	6	0.80	6	1.01	6
	*PGK*	0.03	1	0.01	1	0.28	4	0.50	2
	*β-TUB*	0.09	2	0.02	3	0.18	2	0.51	3
	*UBC*	0.78	6	1.77	7	0.90	7	1.77	7
pHs	*18S*	0.48	6	0.61	7	0.30	4	0.67	7
	*28S*	0.23	3	0.20	3	0.30	3	0.41	4
	*EF1α*	0.11	1	0.18	2	0.38	5	0.38	2
	*GAPDH*	0.41	5	0.49	5	0.13	1	0.60	6
	*PGK*	0.17	2	0.24	4	0.39	6	0.40	3
	*β-TUB*	0.11	1	0.06	1	0.29	2	0.35	1
	*UBC*	0.30	4	0.50	6	0.48	7	0.56	5
Total	*18S*	1.22	5	1.21	5	0.41	2	1.46	5
	*28S*	1.34	6	1.52	7	0.40	1	1.65	7
	*EF1α*	0.55	1	0.93	4	1.38	6	1.24	3
	*GAPDH*	0.55	1	0.65	2	1.23	5	1.15	2
	*PGK*	0.62	2	0.42	1	0.99	4	1.11	1
	*β-TUB*	0.76	3	1.32	6	1.52	7	1.50	6
	*UBC*	0.99	4	0.71	3	0.73	3	1.26	4

Note: M, expression stability value; SV, stability value; CV + SD, coefficient of variation and standard deviation; Avg. SD, average standard deviation.

**Table 3:** Recommended reference genes in *S. rolfsii* under different experimental conditions.

Experimental Conditions	Recommended Reference Genes	
Developmental stages	*GADPH*	*UBC*
Populations	*PGK*	*UBC*
Fungicides	*GAPDH*	*PGK*
Photoperiods	*EF1α*	*PGK*
pHs	*β-TUB*	*EF1α*
All samples	*PGK*	*GAPDH*

## Data Availability

The data presented in this study are available in supplementary material.

## Supplementary Materials

The following supporting information can be downloaded at: https://www.mdpi.com/article/10.3390/ijms242015198/s1.
