# Supplementary material for: Selection and Validation of Reference Genes for Reverse-Transcription Quantitative PCR Analysis in Sclerotium rolfsii
Source: Int J Mol Sci. 2023 Oct 15;24(20):15198. doi: 10.3390/ijms242015198 (PMC10607518; doi:10.3390/ijms242015198)
Supplement: Supplementary file 1 [file ijms-24-15198-s001.zip › ijms-2620527-supplementary.pdf]

Table S1. Expression levels of the seven candidate reference genes.

Gene	All samples	Developmental stage	Population	Fungicides	Photoperiod	pH
18S	6.24±0.61	6.55±0.41	6.33±0.43	5.80±0.61	6.42±0.48	6.71±0.32
28S	8.13±0.44	7.96±0.42	7.97±0.54	7.98±0.29	8.18±0.17	8.66±0.33
EF1α	15.41±1.69	18.08±3.08	15.42±0.83	14.67±0.99	14.74±0.26	15.56±0.42
GAPDH	16.22±1.44	18.53±2.15	16.56±1.04	15.47±0.86	15.59±0.79	16.28±0.18
PGK	18.45±1.33	20.49±2.56	18.46±0.47	18.00±0.71	17.65±0.29	18.52±0.42
β-TUB	18.61±1.90	21.69±3.11	18.17±1.62	17.68±0.89	18.39±0.20	19.01±0.33
UBC	15.68±0.93	16.57±1.68	15.56±0.50	15.44±0.71	15.80±0.90	15.62±0.54

Note: Values are means ± SD.

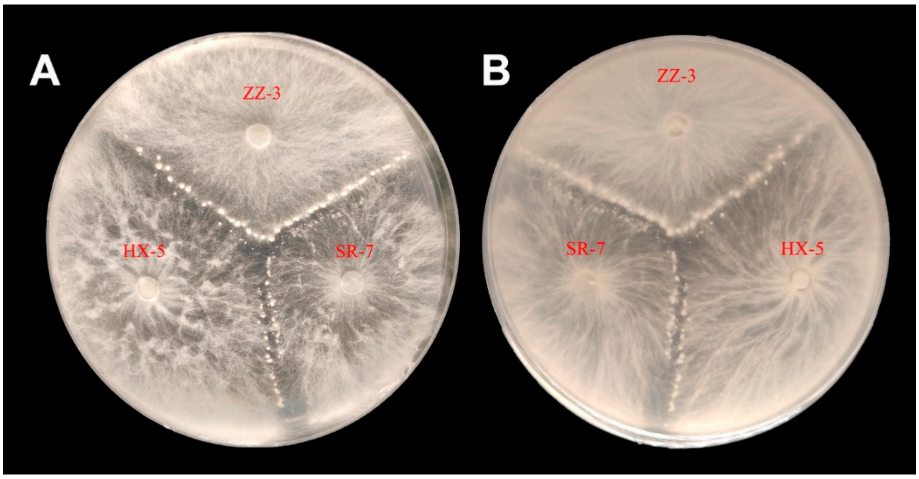

Figure S1. Mycelial interactions of three different mycelial compatibility groups (MCGs). (A) Colony morphology viewed from front side. (B) Colony morphology viewed from reverse side. The diameter of Petri dish is 90 mm.

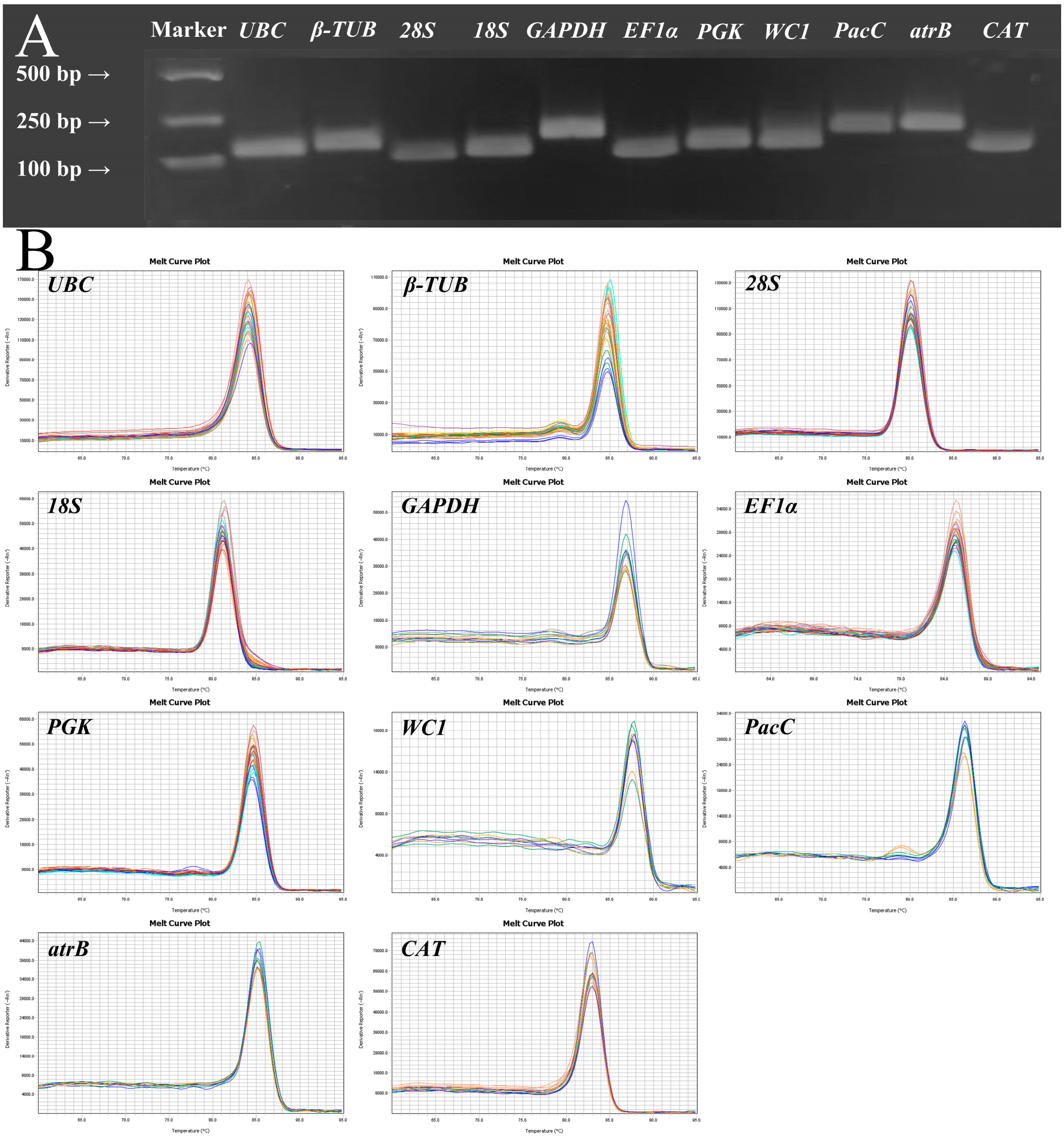

Figure S2. The amplification specificity of designed primers analyzed from (A) 2% agarose gel electrophoresis and (B) melting curve analysis.
